# Propofol and magnesium attenuate isoflurane-induced caspase-3 activation via inhibiting mitochondrial permeability transition pore

**DOI:** 10.1186/2045-9912-2-20

**Published:** 2012-08-17

**Authors:** Yiying Zhang, Yuanlin Dong, Zhipeng Xu, Zhongcong Xie

**Affiliations:** 1 Geriatric Anesthesia Research Unit, Department of Anesthesia, Critical Care and Pain Medicine, Massachusetts General Hospital and Harvard Medical School, 149 13th St. Room 4310, Charlestown, MA, 02129-2060, USA

## Abstract

**Background:**

The inhalation anesthetic isoflurane has been shown to open the mitochondrial permeability transition pore (mPTP) and induce caspase activation and apoptosis, which may lead to learning and memory impairment. Cyclosporine A, a blocker of mPTP opening might attenuate the isoflurane-induced mPTP opening, lessening its ripple effects. Magnesium and anesthetic propofol are also mPTP blockers. We therefore set out to determine whether propofol and magnesium can attenuate the isoflurane-induced caspase activation and mPTP opening.

**Methods:**

We investigated the effects of magnesium sulfate (Mg^2+^), propofol, and isoflurane on the opening of mPTP and caspase activation in H4 human neuroglioma cells stably transfected to express full-length human amyloid precursor protein (APP) (H4 APP cells) and in six day-old wild-type mice, employing Western blot analysis and flowcytometry.

**Results:**

Here we show that Mg^2+^ and propofol attenuated the isoflurane-induced caspase-3 activation in H4-APP cells and mouse brain tissue. Moreover, Mg^2+^ and propofol, the blockers of mPTP opening, mitigated the isoflurane-induced mPTP opening in the H4-APP cells.

**Conclusion:**

These data illustrate that Mg^2+^ and propofol may ameliorate the isoflurane-induced neurotoxicity by inhibiting its mitochondrial dysfunction. Pending further studies, these findings may suggest the use of Mg^2+^ and propofol in preventing and treating anesthesia neurotoxicity.

## Introduction

Alzheimer’s disease (AD) is one of the most common dementia with an incidence of 13% in people over 65 years of age [1]. There are approximately 8.5 million AD patients who will need anesthesia and surgery care every year. Anesthesia and surgery have been reported to induce cognitive dysfunction, which AD patients are susceptible to develop. Therefore, it is important to identify any anesthetic that may promote AD neuropathogenesis and to develop strategies in preventing and treating anesthesia neurotoxicity.

Caspase activation and apoptosis have been reported to contribute to AD neuropathogenesis. ([2-11], reviewed in [12,13]) And current studies suggest that caspase activation (without apoptosis) can induce microglia activation, contributing to AD neuropathogenesis [14]. The commonly used inhalation anesthetic isoflurane has been shown to induce caspase activation and apoptosis, and to increase β-amyloid protein (Aβ) oligomerization and accumulation *in vitro* and *in vivo*[15-23]. Our recent studies have shown that isoflurane can induce mitochondrial dysfunction, e.g., mPTP opening, leading to caspase activation *in vitro* and *in vivo* and impairment of learning and memory function in mice [24]. Moreover, cyclosporine A, an inhibitor of mPTP opening [25-33], has been shown to attenuate the isoflurane-induced mPTP opening, caspase-3 activation, and impairment of learning and memory [24].

Propofol, the most commonly used intravenous anesthetic, and magnesium sulfate (Mg^2+^) are also blockers of mPTP [34]. In the present studies, we have assessed the effects of propofol and Mg^2+^ on isoflurane-induced opening of mPTP and caspase-3 activation.

Both propofol and isoflurane have been shown to be both cytoprotective and cytotoxic, depending on dose- and time-differences in various cell cultures and in the developing brains in different animal models [35-38].Volatile anesthetics or propofol may also provide cardiac or brain protection via opening mitochondrial potassium channels or generation of reactive oxygen species (ROS) in mitochondria [34,39,40].

## Methods

### Cells

We employed H4 human neuroglioma cells, stably transfected to express full-length (FL) amyloid precursor protein (APP) (H4-APP cells) in the experiments. The cells were cultured in Dulbecco's Modified Eagle Media (DMEM) containing 9% heat-inactivated fetal calf serum, 100 units/ml penicillin, 100 μg/ml streptomycin, and 2 mM L-glutamine, and were supplemented with 220 μg/ml G418.

### Treatments for H4-APP cells

**Figure 1:**
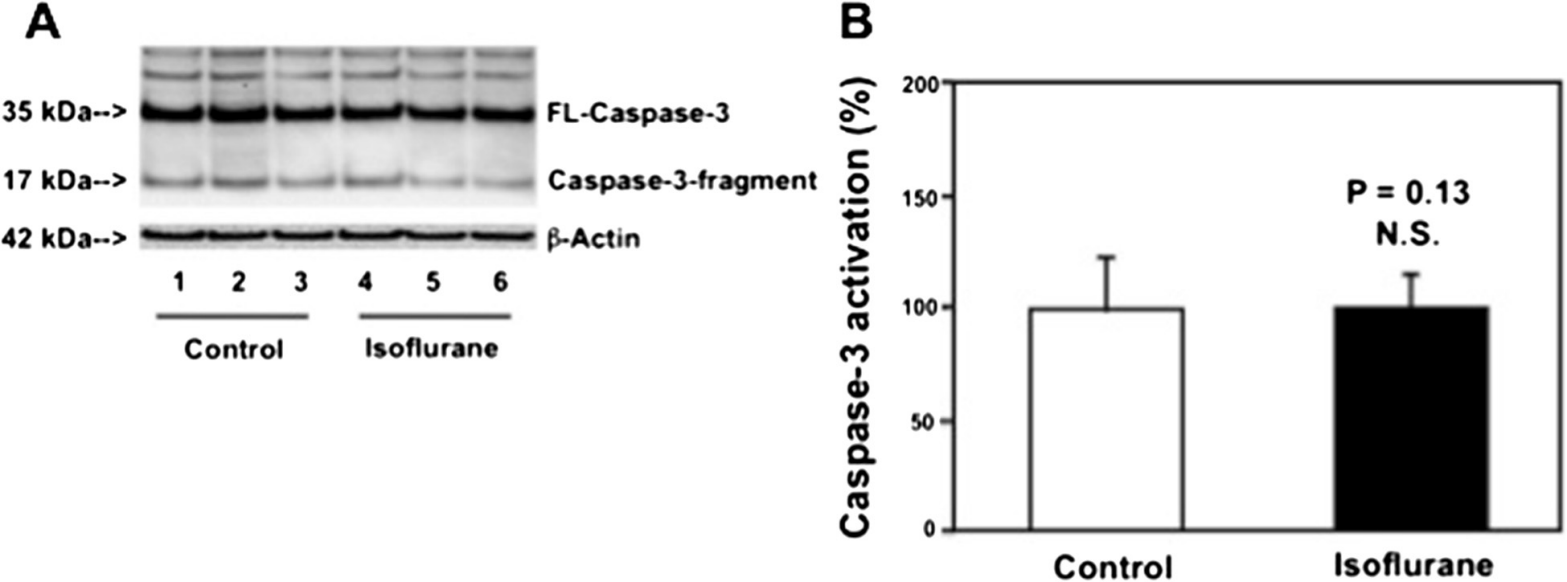
**Isoflurane does not induce caspase-3 activation in H4-APP cells for 3 hours. A**. Western blot shows that treatment of 2% isoflurane for three hours (lanes 4 to 6) does not induce caspase-3 activation as compared to the control condition (lanes 1 to 3) in H4-APP cells. **B**. Quantification of the Western blot shows that isoflurane (black bar) does not induce caspase-3 activation as compared to the control condition (white bar): 0.98 versus 1.00 fold (P = 0.13) in H4-APP cells.

Cells were treated with 2% isoflurane plus 21% O_2_ and 5% CO_2_ for six hours as described by our previous studies [41] for the purpose of measuring caspase-3 activation. The cultured cells were treated for three hours in the studies to measure mPTP opening as described by our precious studies [24]. Treatment with 2% isoflurane for three hours may not induce caspase-3 activation and apoptosis (Figure 1). Thus, we assessed whether the treatment with 2% isoflurane for three hours might induce opening of mPTP without causing caspase-3 activation in the cells. In the interaction experiments, 50 μM magnesium sulfate (Mg^2+^) or 200 μM propofol was administrated to the cells one hour before the isoflurane treatment as well as during isoflurane treatment.

### Mice anesthesia and harvest of brain tissues

C57BL/6 J mice (The Jackson Laboratory, Bar Harbor, ME) were used in the experiments as described before [17,42]. The animal protocol was approved by Standing Committee on Animals at Massachusetts General Hospital (Boston, MA). The mice were randomized by weight and gender into experimental groups that received 1.4% isoflurane plus 100% oxygen for six hours, and control groups that received 100% oxygen for six hours at identical flow rates in identical anesthetizing chambers. Anesthetic and oxygen concentrations were measured continuously (Datex, Tewksbury, MA), and the temperature of the anesthetizing chamber was controlled to maintain the rectal temperature of the mice at 37 ± 0.5°C. In the interaction studies, Mg^2+^ (100 mg/kg) or propofol (50 mg/kg) was administered to the mice via intraperitoneal injection 10 minutes before the isoflurane anesthesia. 200 μM propofol has been shown to have neuroprotective effects in an *in vitro* model of traumatic brain injury[43]; we therefore used this concentration of propofol to determine whether propofol can attenuate the isoflurane-induced mPTP opening. 50 and 100, but not 25, mg/kg propofol have been shown to produce neuroprotection effects in ischemic mice models [44]. Thus, we used 50 mg/kg propofol in the current studies. And we used 100 mg/kg Mg^2+^ on mice because Mg^2+^ has been shown to have a neuroprotective effect on cerebral ischemia [45]. And based on our preliminary results, we used 50 μM Mg^2+^ in the *in vitro* the studies. Whole brain tissues of mice were harvested at end of the anesthesia.

### Brain tissue lysis and protein amount quantification

The harvested brain tissues were homogenized on ice using an immunoprecipitation buffer (10 mM Tris–HCl, pH 7.4, 150 mM NaCl, 2 mM EDTA, 0.5% Nonidet P-40) plus protease inhibitors (1 μg/ml aprotinin, 1 μg/ml leupeptin, 1 μg/ml pepstatin A). The lysates were collected, centrifuged at 13,000 rpm for 15 min, and quantified for total proteins by a bicinchoninic acid protein assay kit (Pierce, Iselin, NJ).

### Western blots analysis

The harvested H4-APP cells and brain tissues were subjected to Western blot analyses as described by Xie et al. [17,18,20] and Zhang et al. [46,47]*.* A caspase-3 antibody (1:1,000 dilution; Cell Signaling Technology, Inc., Danvers, MA) was used to recognize FL-caspase-3 (35 – 40 kDa) and caspase-3 fragment (17 – 20 kDa) resulting from cleavage at asparate position 175. Antibody anti-β-Actin (1:10,000, Sigma, St. Louis, MO) was used to detect β-Actin (42 kDa). Each band in the Western blot represented an independent experiment. The results were averaged from three to 8 independent experiments. Briefly, the intensity of the signals was analyzed using the National Institute of Health image program (National Institute of Health Image 1.62, Bethesda, MD). The caspase-3 normalization was performed by determining the ratio of caspase-3 fragment to FL caspase-3. Then, the changes in levels of caspase-3 in treated cells or mice were presented as percentages of the corresponding levels in control cells or mice.

### Flow cytometric analysis of mPTP opening

H4-APP cells were treated with 2% isoflurane for three hours. For the interaction studies, 50 μM Mg^2+^ or 200 μM propofol was administrated to cells one hour before the isoflurane treatment. The opening of mPTP was determined by flowcytometry, using the MitoProbe^TM^ Transition Pore Assay Kit (Invitrogen, Carlsbad, CA). In normal conditions, the non-fluorescent acetoxymethyl ester (AM) of calcein dye (calcein AM) and cobalt can enter the cell. The acetoxymethyl ester (AM) groups are cleaved from calcein via non-specific esterase, and calcein can then show fluorescence signals in both the cytosol and mitochondria. Cobalt can quench the cytosolic calcein signal. However, cobalt cannot enter healthy mitochondria freely, and therefore cannot quench the mitochondrial calcein signal. When opening of mPTP occurs, cobalt enters through the pore and subsequently quenches the mitochondrial calcein signal. Flowcytometry was used to detect the amount of cells that exhibit quenched calcein signals inside the mitochondria. The location of the curves indicates the amount of such cells, which suggests the opening of mPTP. Ionomycin was used as a positive control for the opening of mPTP in the experiments. Dead cells and debris were excluded from analysis by gates set on forward and side angle light scatter.

### Statistics

Given the presence of background caspase 3 activation in cells and brain tissues of mice, we did not use absolute values to describe these changes. Instead, these changes were presented as percentages of those from the control group. For example, one hundred percent of caspase-3 activation refers to the control level for the purpose of comparison to experimental conditions. Data were expressed as mean ± S.D. The number of samples varied from three to 8, and the samples were normally distributed. We used a two-tailed *t*-test to compare the difference between the control condition and isoflurane treatment, and the difference between propofol, Mg^2+^ and their controls. P-values less than 0.05 (* or #) and 0.01 (** or ##) were considered statistically significant.

## Results

### Mg^2+^ inhibited the isoflurane-induced caspase-3 activation in H4-APP cells and in brain tissues of mice

**Figure 2:**
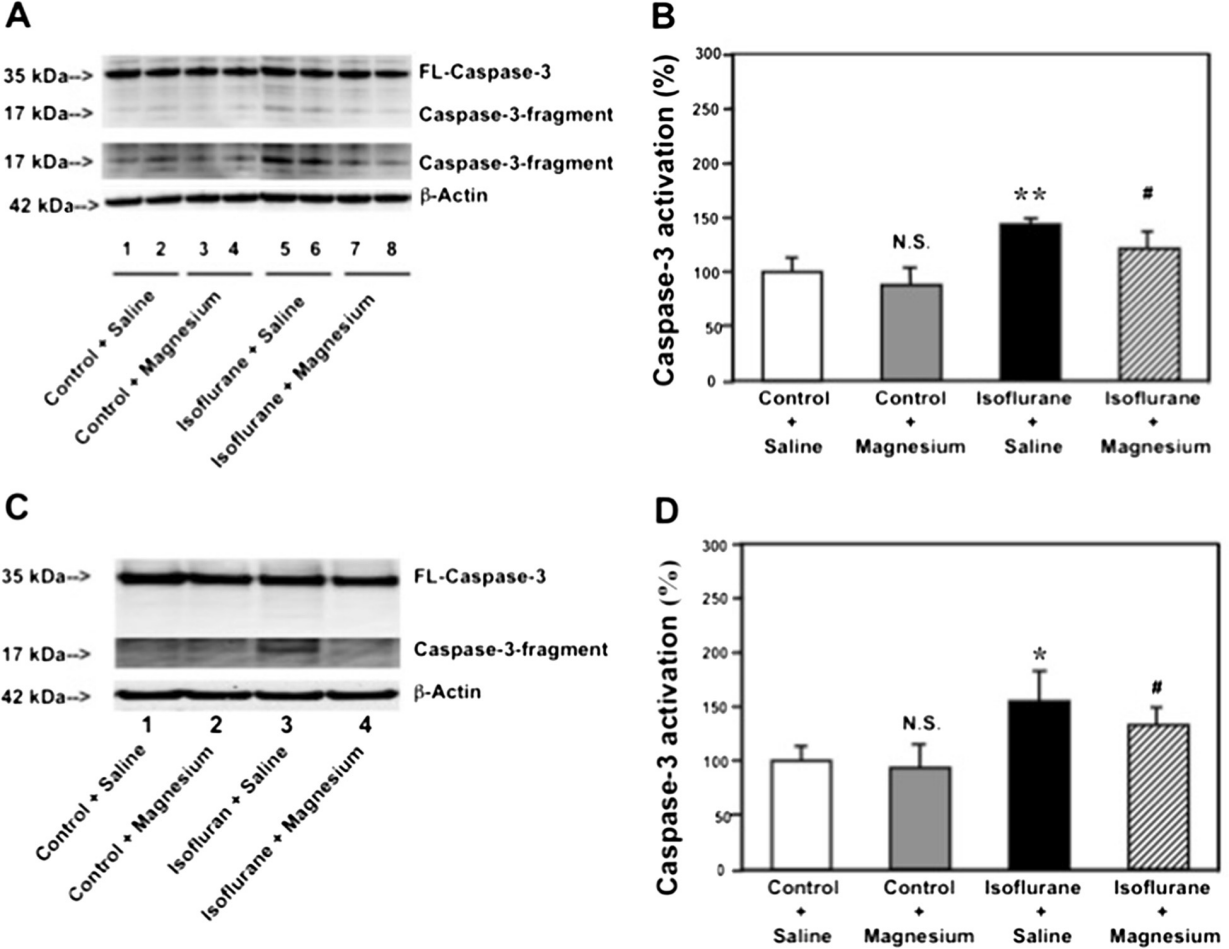
**Mg**^**2+**^**attenuates isoflurane-induced caspase-3 activation in H4-APP cells and brain tissues of mice. A**. Western blot shows that treatment of 2% isoflurane for six hours (lanes 5 and 6) induces caspase-3 activation as compared to the control condition (lanes 1 and 2) in H4-APP cells. Mg^2+^ treatment alone (lanes 3 and 4) does not induce caspase-3 activation as compared to the control condition (lanes 1 and 2), but Mg^2+^ treatment attenuates isoflurane-induced caspase-3 activation (lanes 7 and 8) as compared to isoflurane treatment (lanes 5 and 6) in H4-APP cells. **B**. Quantification of the Western blot shows that isoflurane (black bar) induces caspase-3 activation as compared to the control condition (white bar): 1.54 versus 1.00 fold (** P = 0.001) in H4-APP cells. Mg^2+^ treatment (net bar) attenuates isoflurane-induced caspase-3 activation as compared to isoflurane treatment (black bar): 1.23 fold versus 1.54 fold (# P = 0.03) in H4-APP cells. **C**. Western blots shows that treatment of 1.4% isoflurane for six hours (lane 3) induces caspase-3 activation as compared to the control condition (lane 1) in mouse brain tissues. Mg^2+^ treatment alone (lane 2) does not induce caspase-3 activation as compared to the control condition (lane 1), but Mg^2+^ treatment attenuates isoflurane-induced caspase-3 activation (lane 4) as compared to isoflurane treatment (lane 3) in mouse brain tissues. **D**. Quantification of the Western blot shows that isoflurane (black bar) induces caspase-3 activation as compared to the control condition (white bar): 1.52 versus 1.00 fold (* P = 0.02) in mouse brain tissues. Mg^2+^ treatment (net bar) attenuates isoflurane-induced caspase-3 activation as compared to isoflurane treatment (black bar): 1.38 versus 1.52 fold (# P = 0.03) in mouse brain tissues.

The H4-APP cells were treated with 50 μM Mg^2+^ or saline for 10 minutes followed by 2% isoflurane or control condition for six hours. The cells were harvested at the end of the experiment and were subjected to Western blot analysis. Caspase-3 immunoblotting revealed that the isoflurane treatment induced caspase-3 activation (Figure 2A) as evidenced by increased ratios of cleaved (activated) caspase-3 fragment (17 kDa) to full-length (FL) (35–40 kDa) caspase-3. Treatment with 50 μM Mg^2+^ alone did not induce caspase-3 activation, but the Mg^2+^ treatment attenuated the isoflurane-induced caspase-3 activation (Figure 2A). Quantification of the Western blots (Figure 2B), based on the ratio of caspase-3 fragment to FL caspase-3, revealed that isoflurane (black bar) led to caspase-3 activation as compared to the control condition (white bar): 1.54 versus 1.00 fold (** P = 0.001). The Mg^2+^ treatment (net bar) attenuated the isoflurane-induced caspase-3 activation: 1.23 fold versus 1.54 fold (# P = 0.03). These findings suggest that Mg^2+^ may mitigate the isoflurane-induced caspase-3 activation in H4-APP cells.

Next, we performed the *in vivo* relevance studies by assessing the effects of isoflurane and Mg^2+^ on caspase-3 activation in the brain tissues of six-day old WT mice. As can be seen in Figure 2C, Mg^2+^ (lane 4) attenuated the isoflurane-induced caspase-3 activation (lane 3) in the brain tissues of the mice. The Mg^2+^ treatment alone (lane 2) did not induce caspase-3 activation as compared to the saline group (lane 1) in the brain tissues of the mice. Quantification of the Western blot further illustrated that the isoflurane (black bar) led to caspase-3 activation as compared to the control condition (white bar): 1.52 versus 1.00 fold (* P = 0.02). Mg^2+^ treatment (net bar) attenuated the isoflurane-induced caspase-3 activation (black bar): 1.38 versus 1.52 fold (# P = 0.03), (Figure 2D). These results from the *in vivo* studies further suggest that Mg^2+^ may attenuate the isoflurane-induced caspase-3 activation.

### Propofol inhibited isoflurane-induced caspase-3 activation in brain tissues of mice

**Figure 3:**
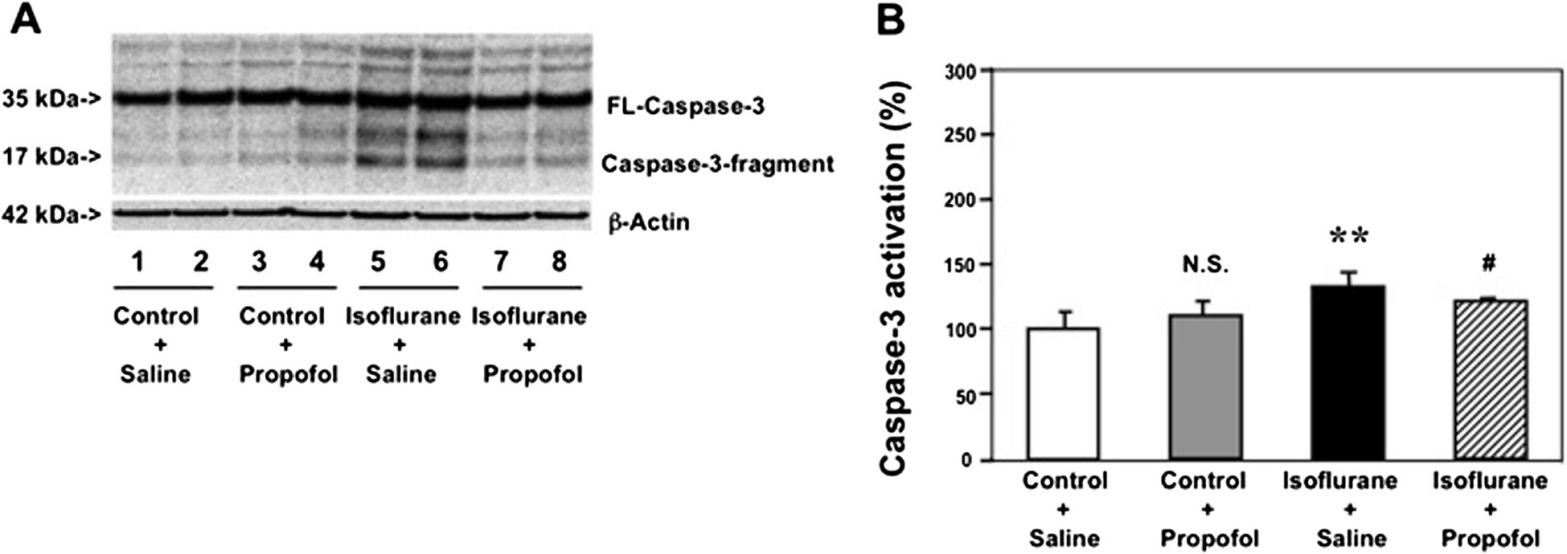
**Propofol attenuates isoflurane-induced caspase-3 activation in brain tissues of mice. A**. Western blot shows that treatment of 1.4% isoflurane for six hours (lane 7–9) induces caspase-3 activation as compared to the control condition (lane 1–3). Propofol treatment alone (lane 4–6) does not induce caspase-3 activation as compared to the control condition (lane 1–3), but propofol treatment attenuates isoflurane-induced caspase-3 activation (lane 11–12) as compared to isoflurane treatment (lane 7–9). **B**. Quantification of the Western blot shows that isoflurane (black bar) induces caspase-3 activation as compared to the control condition (white bar): 1.33 versus 1.00 fold (** P = 0.01). Propofol treatment (net bar) attenuates isoflurane-induced caspase-3 activation as compared to isoflurane treatment (black bar): 1.20 versus 1.33 fold (# P = 0.02) in mouse brain tissues.

Our previous studies have illustrated that propofol can attenuate the isoflurane-induced caspase-3 activation in H4-APP cells. [48]. In the current experiments, we performed the *in vivo* relevance studies by assessing the effects of isoflurane and propofol on caspase-3 activation in the brain tissues of WT six-day old mice. As can be seen in Figure 3A, propofol (lane 10–11) attenuated the isoflurane-induced caspase-3 activation (lane 7–9) in the brain tissues of the mice. The propofol treatment alone (lane 4–6) did not induce caspase-3 activation compared with the saline group (lane 1–3) in the brain tissues of the mice. Quantification of the Western blot further illustrated that the isoflurane anesthesia (black bar) led to caspase-3 activation as compared to the control condition (white bar): 1.33 versus 1.00 fold (** P = 0.01). Propofol treatment (net bar) attenuated the isoflurane-induced caspase-3 activation in the mice (black bar): 1.20 versus 1.33 fold (# P = 0.02), (Figure 3B). These results from the *in vivo* studies further suggest that propofol may attenuate the isoflurane-induced caspase-3 activation.

### Mg^2+^ and propofol inhibit isoflurane-induced opening of mPTP

Given that Mg^2+^ and propofol can attenuate the isoflurane-induced caspase-3 activation, and the isoflurane-induced caspase-3 activation may result from the isoflurane-induced opening of mPTP, next, we asked whether Mg^2+^ and propofol, the blockers of mPTP opening, can attenuate the isoflurane-induced mPTP opening.

**Figure 4:**
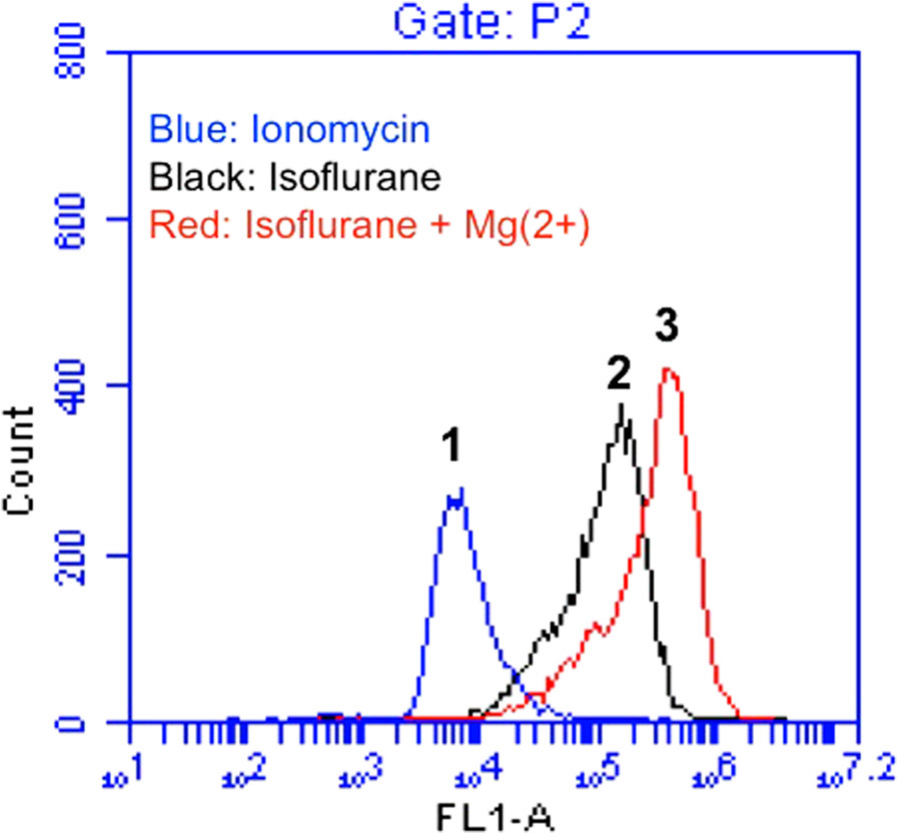
**Mg**^**2+**^**attenuates isoflurane-induced opening of mPTP in H4-APP cells. A.** Flow cytometric analysis shows changes in calcein levels in mitochondria of H4-APP cells stained with calceinAM or calceinAM plus cobalt, which indicate the opening of mPTP. Peak 1: treatment of ionomycin (the positive control of opening of mPTP); peak 2: treatment of isoflurane; peak 3: treatment of isoflurane plus Mg^2+^ (50 μM). Mg^2+^ treatment attenuates isoflurane-induced opening of mPTP, as demonstrated by the position of peak of isoflurane treatment is shifted to the right following Mg^2+^ treatment.

**Figure 5:**
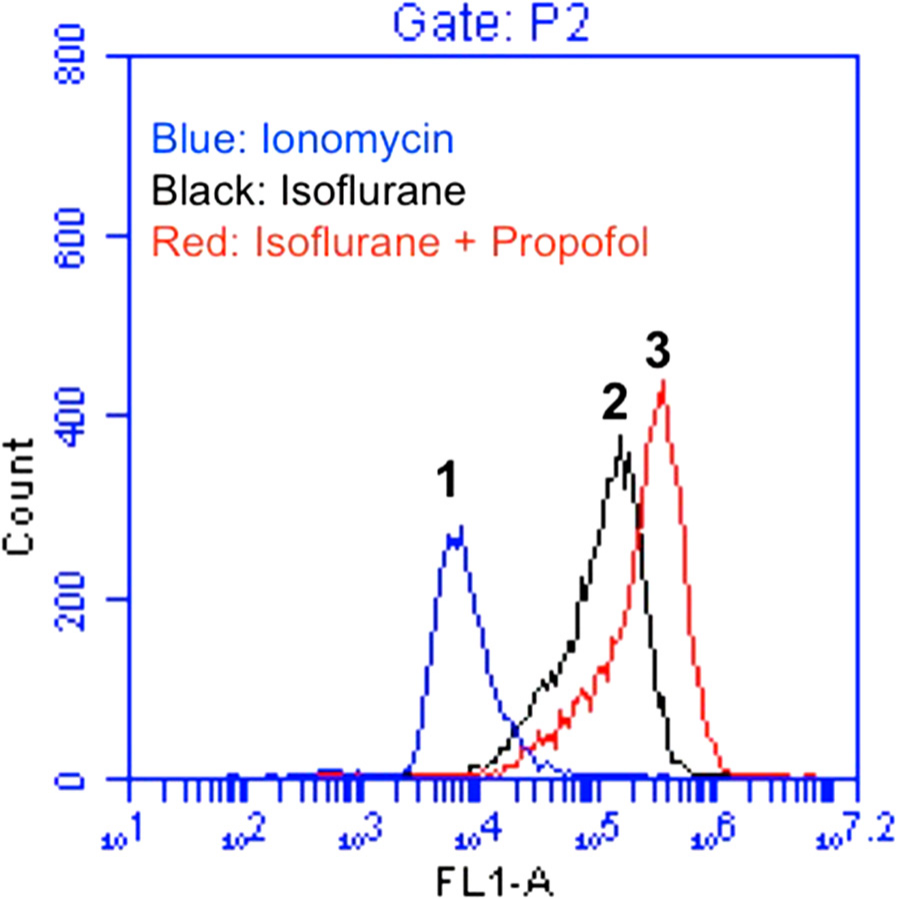
**Propofol attenuates isoflurane-induced opening of mPTP in H4-APP cells. A.** Flow cytometric analysis shows changes in calcein levels in mitochondria of H4-APP cells stained with calceinAM or calceinAM plus cobalt, which indicate the opening of mPTP. Peak 1: treatment of ionomycin (the positive control of opening of mPTP); peak 2: treatment of isoflurane; peak 3: treatment of isoflurane plus propofol (200 μM). Propofol treatment attenuates isoflurane-induced opening of mPTP, as demonstrated by the position of peak of isoflurane treatment is shifted to the right following propofol treatment.

Flow cytometric analysis of calceinAM and cobalt showed that the treatment with 50 μM Mg^2+^ (Figure 4, peak 3) led to reductions in the isoflurane-induced mPTP opening (Figure 4, peak 2), as evidenced by the right-shift of the curve, whereas the Mg^2+^ treatment alone did not affect the opening of mPTP in H4-APP cells (data not shown). Next, we found that the treatment with 50 μM propofol (Figure 5, peak 3) led to reductions in the isoflurane-induced mPTP opening (Figure 5, peak 2), whereas the propofol treatment alone did not affect the opening of mPTP in H4-APP cells (data not shown). Taken together, these findings suggested that Mg^2+^ and propofol may mitigate the isoflurane-induced caspase-3 activation by inhibiting the isoflurane-induced opening of mPTP.

## Discussion

Previous studies have shown that the common inhalation anesthetic isoflurane may induce neurotoxicity *in vitro*[18] and *in vivo*[17], which may lead to learning and memory impairment in mice [24,49,50] and cognitive dysfunction in humans [24]. In our search for the strategy to prevent and treat isoflurane neurotoxicity, we were able to show that mPTP inhibitor CsA could attenuate the isoflurane-induced mitochondrial dysfunction (e.g., inhibition of mPTP) and caspase-3 activation. However, CsA is not routinely used in patients due to its nephrotoxicity, hepatotoxicity and cardiotoxicity side effect [51]. Therefore, it is important to assess whether other mPTP inhibitors can also attenuate the isoflurane-induced neurotoxicity.

We have found that both propofol and Mg^2+^, two chemicals with no significant side effects, can attenuate the isoflurane-induced caspase-3 activation *in vitro* and in the brain tissues of mice (Figures 2 and 3). These data suggest that propofol and Mg^2+^ may attenuate the isoflurane-induced neurotoxicity.

**Figure 6:**
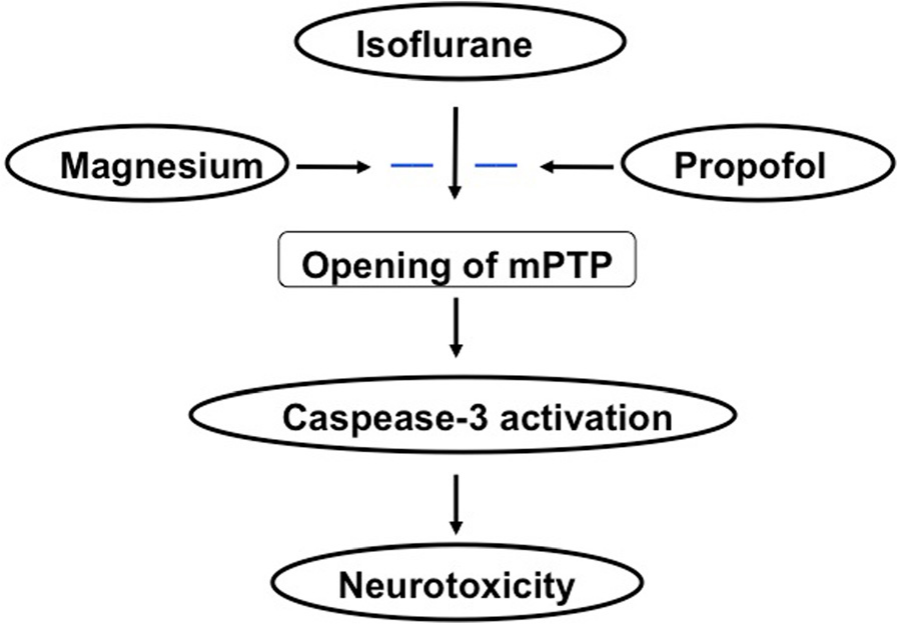
**Hypothetical pathway by which Propofol and magnesium attenuate isoflurane-induced cytotoxicity.** Propofol and magnesium may mitigate the isoflurane-induced caspase-3 activation by inhibiting the isoflurane-induced mPTP opening.

For the mechanistic studies, we have shown that both Mg^2+^ (Figure 4) and propofol (Figure 5) can inhibit the isoflurane-induced mPTP opening. Our previous studies have revealed that isoflurane may induce caspase activation, apoptosis, and learning and memory impairment by inducing mitochondrial dysfunction (e.g., mPTP opening) [24,46]. Collectively, These findings suggest that propofol and magnesium may mitigate the isoflurane-induced caspase-3 activation by inhibiting the isoflurane-induced mPTP opening, pending on further studies (Figure 6).

The studies have a few limitations. First, we did not assess whether Mg^2+^ and propofol can ameliorate the isoflurane-induced learning and memory impairment. However, the findings from the current studies showed that Mg^2+^ and propofol inhibit the isoflurane-induced mitochondrial dysfunction and neurotoxicity would establish a system for future studies in animals and in humans. Second, we only measured caspase-3 activation in current studies. This is because our previous studies have already shown that isoflurane can induce caspase-3 activation, apoptosis, Aβ accumulation, and neuroinflammation [17,18,20,52]. In addition, a recent study by Burguillos et al. [14] has shown that caspase activation alone without apoptosis may still be able to contribute to AD neuropathogenesis. Meanwhile, Mg^2+^ is a well-known NMDA receptor antagonist [53]. Isoflurane has been shown to induce neurotoxicity by increased activation of the NMDA receptor [41]. Therefore, it cannot be excluded that Mg^2+^ may inhibit the isoflurane-induced neurotoxicity by inhibiting its effects on the NMDA receptor. Isoflurane may induce neurotoxicity via ROS generation [46,54] and potassium channel activity [55]. Propofol may also affect ROS generation and potassium channel activity in mitochondria [56,57]. Thus, it is also possible that propofol may mitigate the isoflurane-induced caspase activation through ROS and potassium channel activity.

In conclusion, we have found that Mg^2+^ and propofol can attenuate commonly used inhalation anesthetic isoflurane-induce caspase-3 activation *in vitro* and *in vivo*. Furthermore, we have found that Mg^2+^ and propofol, the blockers of mPTP opening, can attenuate isoflurane-induced opening of mPTP.

Our current findings should lead to additional studies to determine the potential effects of anesthetics on AD neuropathogenesis, the underlying mechanisms, and the strategy for prevention and treatment. Ultimately, these combined efforts of anesthesia and neurology may develop guidelines regarding how to provide safer anesthesia care for AD patients (e.g., to avoid worsening of AD neuropathogenesis and decline of cognitive function by anesthesia and surgery), like the one developed by combined efforts of anesthesia and cardiology on safer anesthesia care for coronary artery disease patients.

## Abbreviations

AD: Alzheimer’s disease; APP: Amyloid β precursor protein; Aβ: β-amyloid protein; SD: Standard deviation; SEM: Standard error of mean; CsA: Cyclosporine A; mPTP: Mitochondrial permeability transition pores; calcein AM: Calcein acetoxymethyl ester.

## Competing interests

The authors have no conflicts of interest for the study.

## Authors’ contributions

YZ Study concept and design, Acquisition of data, Analysis and interpretation of data, Drafting of the manuscript, Critical revision of the manuscript for important intellectual content. YD and ZX Analysis and interpretation of data. ZX Obtained funding, Study concept and design, Analysis and interpretation of data, Drafting of the manuscript, Critical revision of the manuscript for important intellectual content, Study supervision. All authors read and have approved the manuscript.
